# Supplementary material for: RTN4IP1 is required for the final stages of mitochondrial complex I assembly and CoQ biosynthesis
Source: EMBO J. 2025 Aug 26;44(19):5482–508. doi: 10.1038/s44318-025-00533-x (PMC12489013; doi:10.1038/s44318-025-00533-x)
Supplement: Supplementary file 1 — Table EV1 [file 44318_2025_533_MOESM1_ESM.docx]

**Expanded View Tables**

**Table EV1**

Plasmids and oligonucleotides used in this study:

| **Plasmid** | **Description** | **Use** |
| --- | --- | --- |
| pLVX-AcGFP1-N1 | GFP in pLVX | Cell line generation |
| pLVX-AcGFP1-N1-RTN4IP1-FLAG | RTN4IP1-Flag in pLVX | Cell line generation |

| **Primer** | **Sequence (5’ to 3’)** | **Use** |
| --- | --- | --- |
| pLVX F | AAGACGATGACGACAAGTAAtgagcggccgcgactctaga | Gibson assembly |
| pLVX R | CAAGTCTTCAGAAATTCCATgaccggtggatcccgggccc | Gibson assembly |
| RNT4IP1-FLAG F | accgcgggcccgggatccaccggtcATGGAATTTCTGAAGACTTG | Gibson assembly |
| RTN4IP1-FLAG R | aattatctagagtcgcggccgctcaTTACTTGTCGTCATCGTCTT | Gibson assembly |
