# Supplementary figures and images for: RTN4IP1 is required for the final stages of mitochondrial complex I assembly and CoQ biosynthesis
Source: EMBO J. 2025 Aug 26;44(19):5482–508. doi: 10.1038/s44318-025-00533-x (PMC12489013; doi:10.1038/s44318-025-00533-x)

Figure 1D

Figure panel:

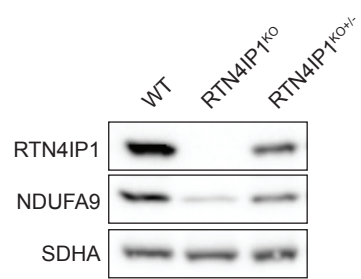

Raw images:

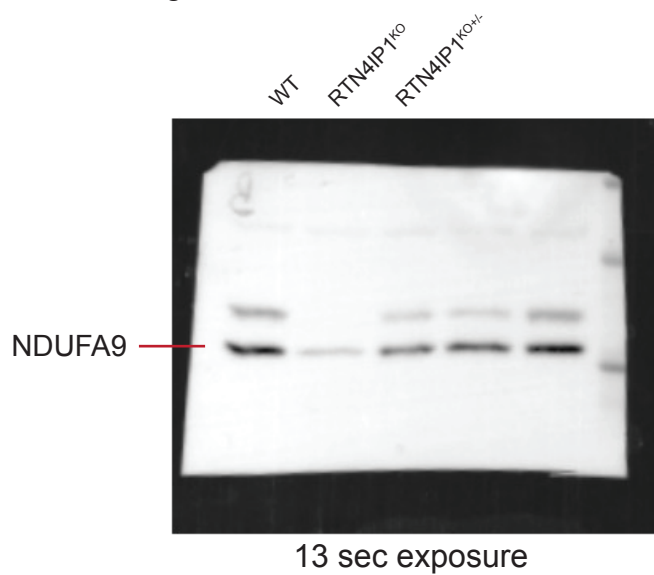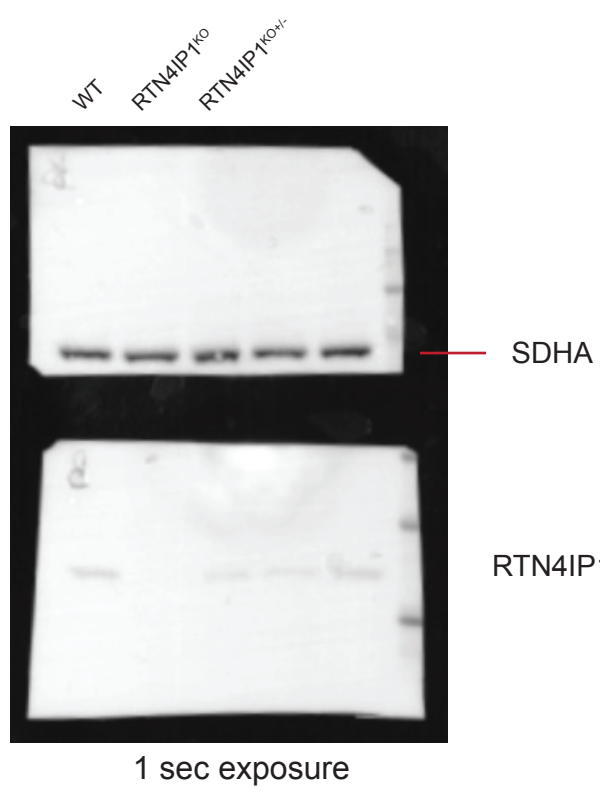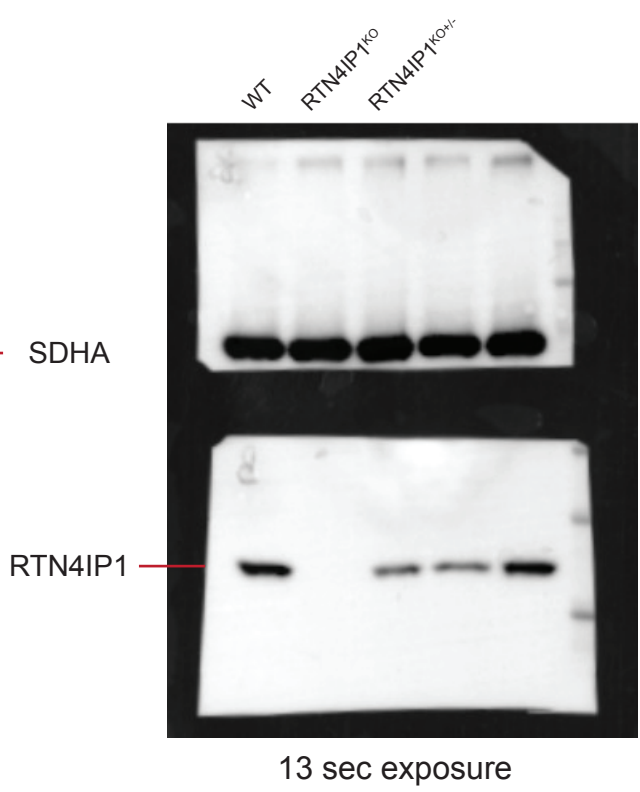

Supplement: Supplementary file 3 — Source data Fig. 1 [file 44318_2025_533_MOESM3_ESM.zip › Source Data Figure 1/SD Figure 1D.pdf]

Figure 1E

Figure panel:

Raw images:

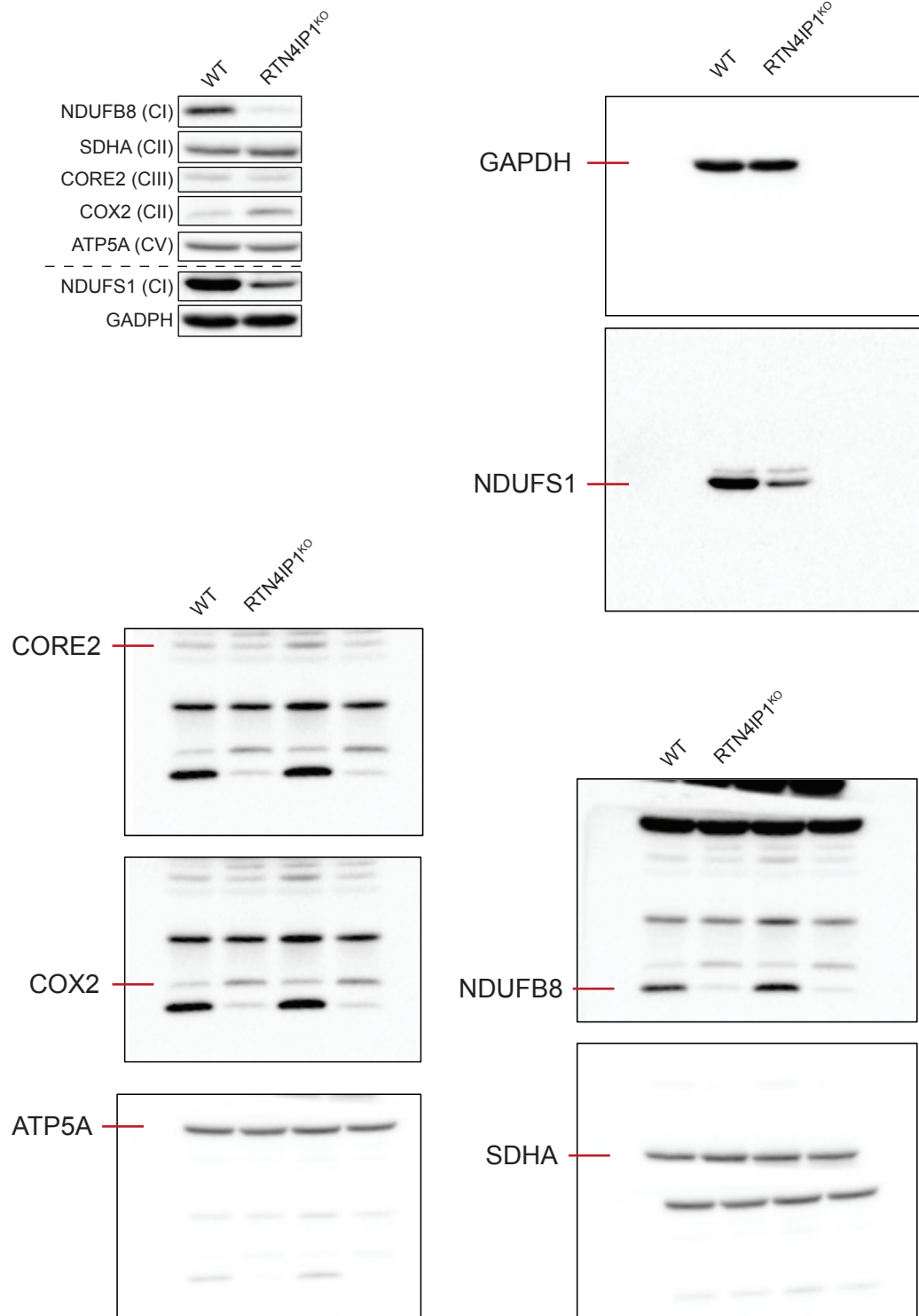

Supplement: Supplementary file 3 — Source data Fig. 1 [file 44318_2025_533_MOESM3_ESM.zip › Source Data Figure 1/SD Figure 1E.pdf]

Figure 2B

Figure panel:

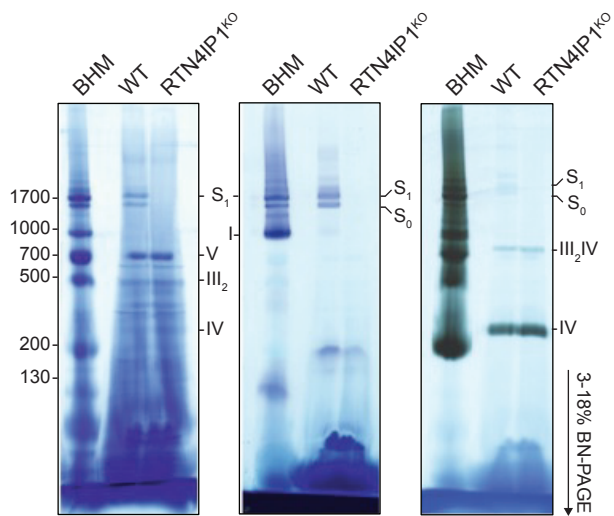

Raw images:

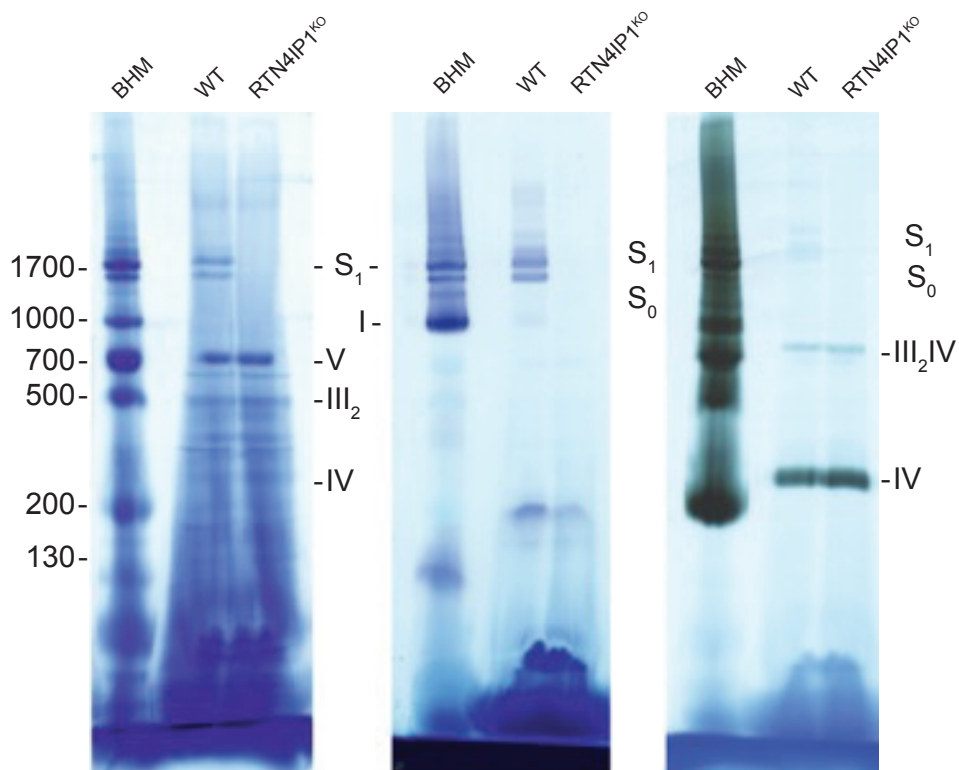

Supplement: Supplementary file 4 — Source data Fig. 2 [file 44318_2025_533_MOESM4_ESM.zip › Source Data Figure 2/SD Figure 2B.pdf]

Figure 2C

Figure panel:

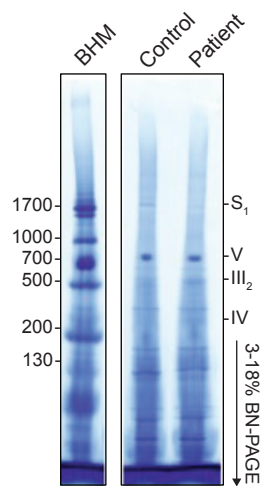

Raw images:

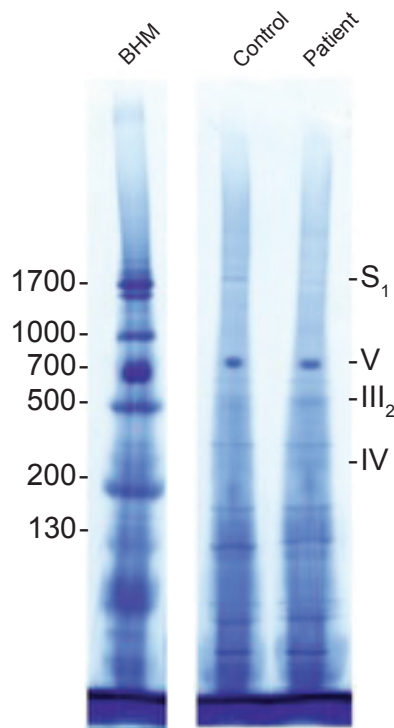

Supplement: Supplementary file 4 — Source data Fig. 2 [file 44318_2025_533_MOESM4_ESM.zip › Source Data Figure 2/SD Figure 2C.pdf]

Figure 2A

Figure panel:

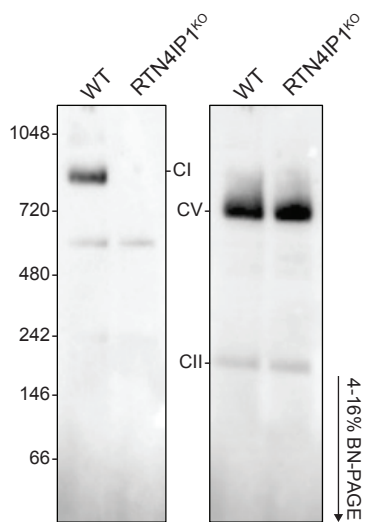

Raw images:

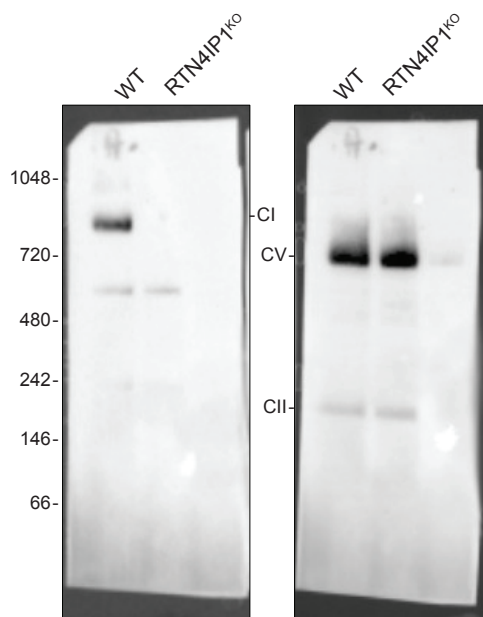

Supplement: Supplementary file 4 — Source data Fig. 2 [file 44318_2025_533_MOESM4_ESM.zip › Source Data Figure 2/SD Figure 2A.pdf]

Figure 5C

Figure panel:

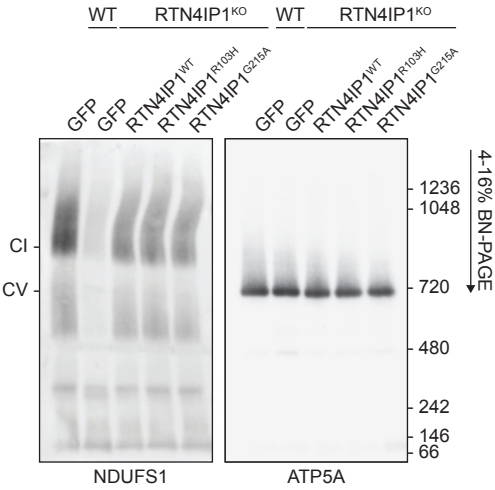

Raw images:

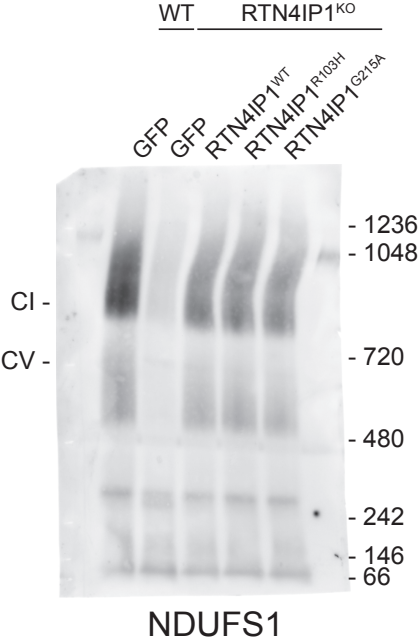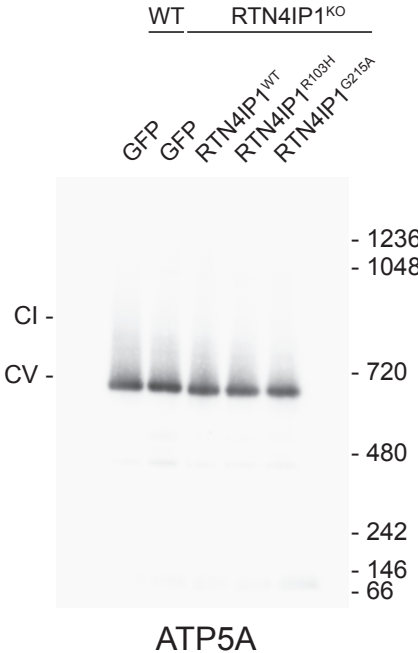

Supplement: Supplementary file 5 — Source data Fig. 5 [file 44318_2025_533_MOESM5_ESM.zip › Source Data Figure 5/SD Figure 5C.pdf]

Figure EV5 A

Figure panel:

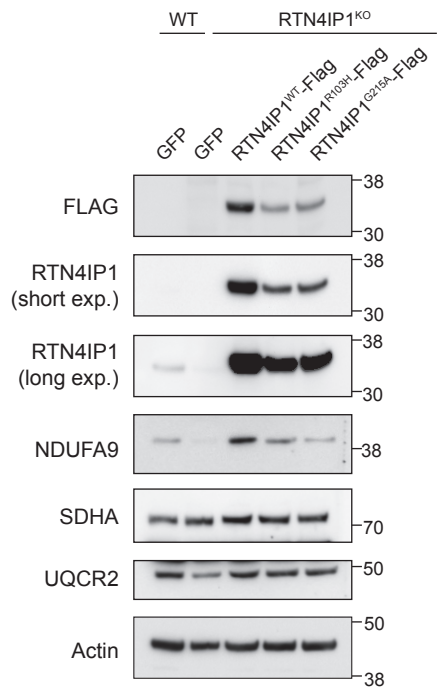

Raw images:

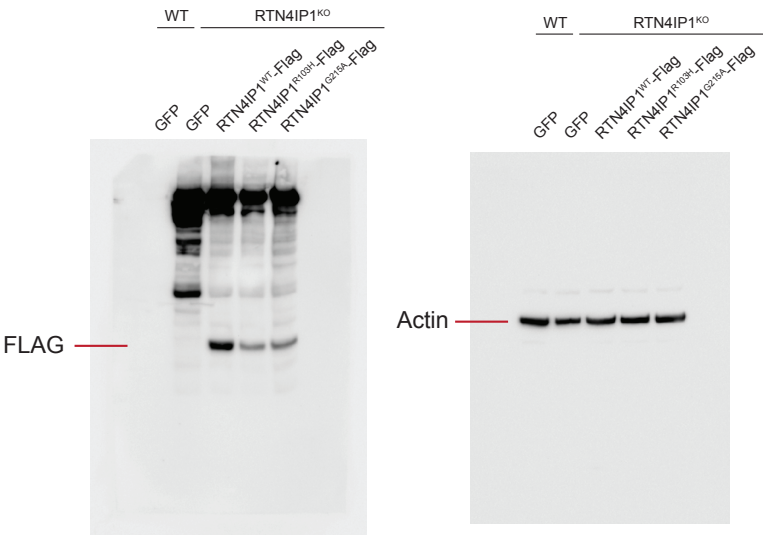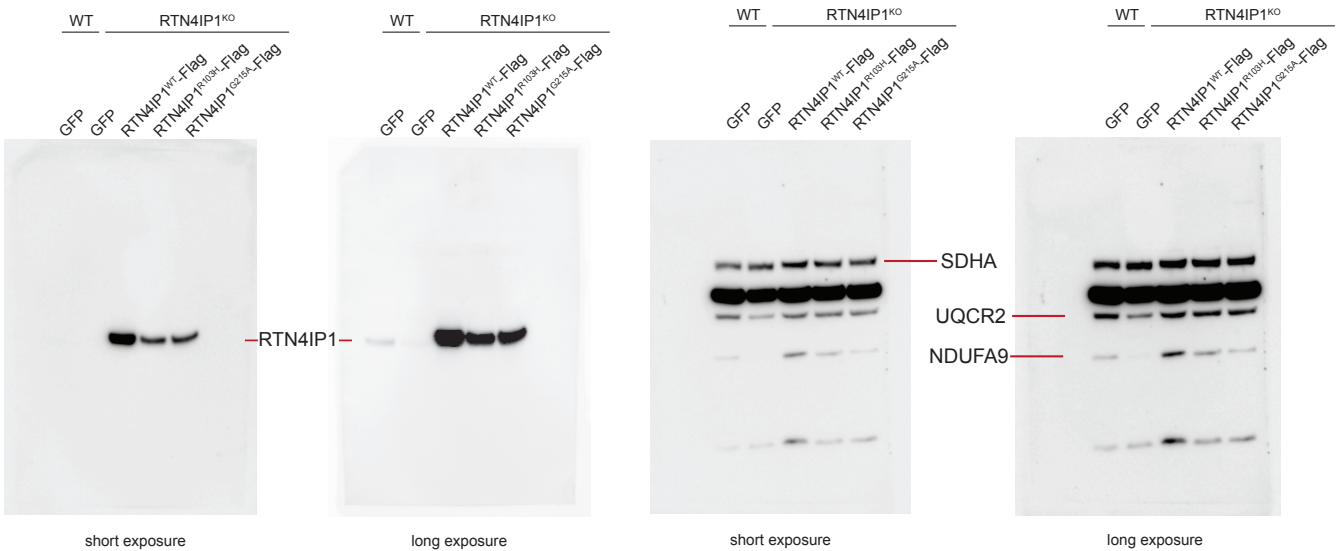

Supplement: Supplementary file 6 — EV Figure Source Data [file 44318_2025_533_MOESM6_ESM.zip › Source Data EV Figures/SD EV Figures.pdf]
