# Supplementary material for: RTN4IP1 is required for the final stages of mitochondrial complex I assembly and CoQ biosynthesis
Source: EMBO J. 2025 Aug 26;44(19):5482–508. doi: 10.1038/s44318-025-00533-x (PMC12489013; doi:10.1038/s44318-025-00533-x)
Supplement: Supplementary file 7 — Expanded View Figures [file 44318_2025_533_MOESM7_ESM.pdf]

Expanded View Figures

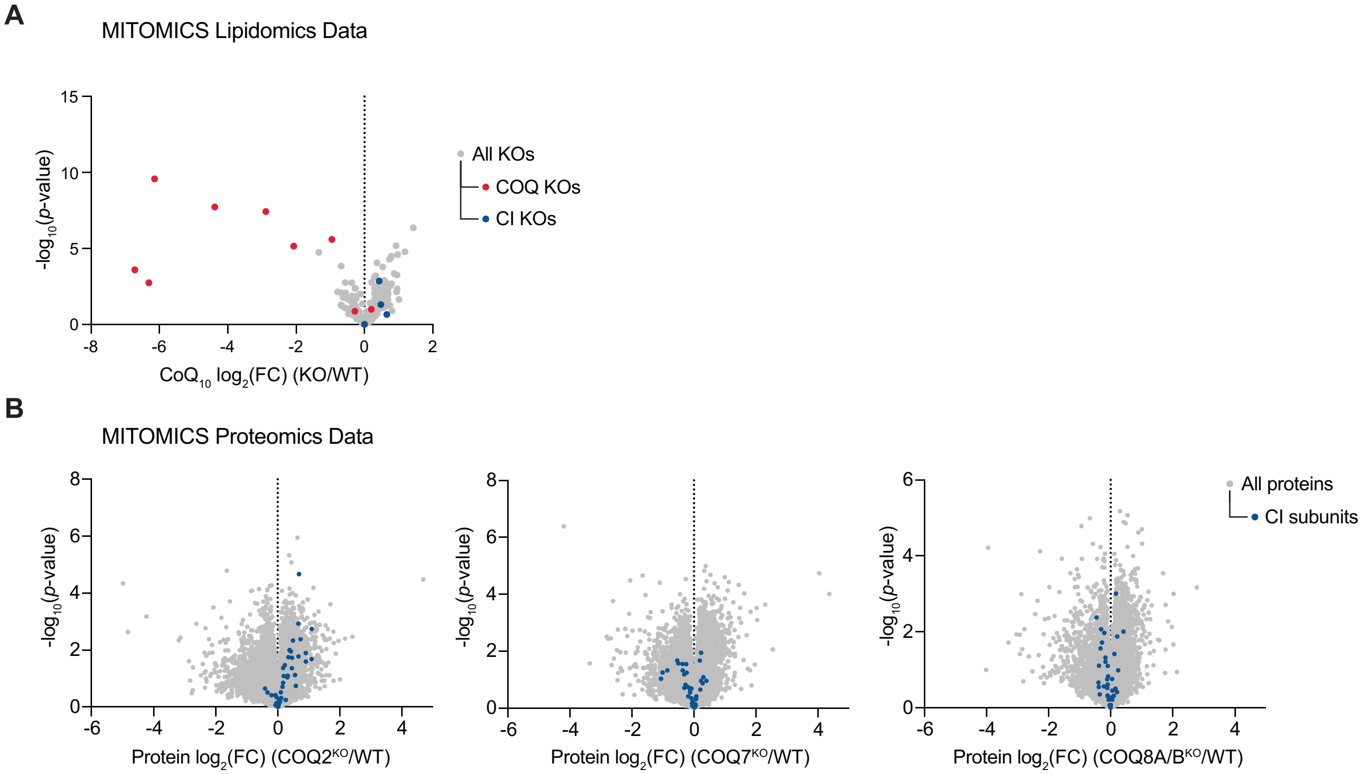

Figure EV1. Perturbations in CI and COQ proteins do not lead to interdependent phenotypes.

(**A**, **B**) Targeted lipidomics for CoQ_{10} abundance (**A**) and proteomics (**B**) analyses from the MITOMICS study (Rensvold et al, 2022) investigating the reciprocal interaction between COQ and CI proteins. Volcano plots depict the $\log_2$ fold change of CoQ_{10} (**A**) or protein (**B**) abundance of all HAP1 knock out cell lines relative to WT. In (**A**), COQ KO cell lines are highlighted in red, CI KO cell lines are highlighted in blue. In (**B**), CI subunits are highlighted in blue. Data shown as mean ($n = 3$), two-sided Student's *t*-test.

 © The Author(s)

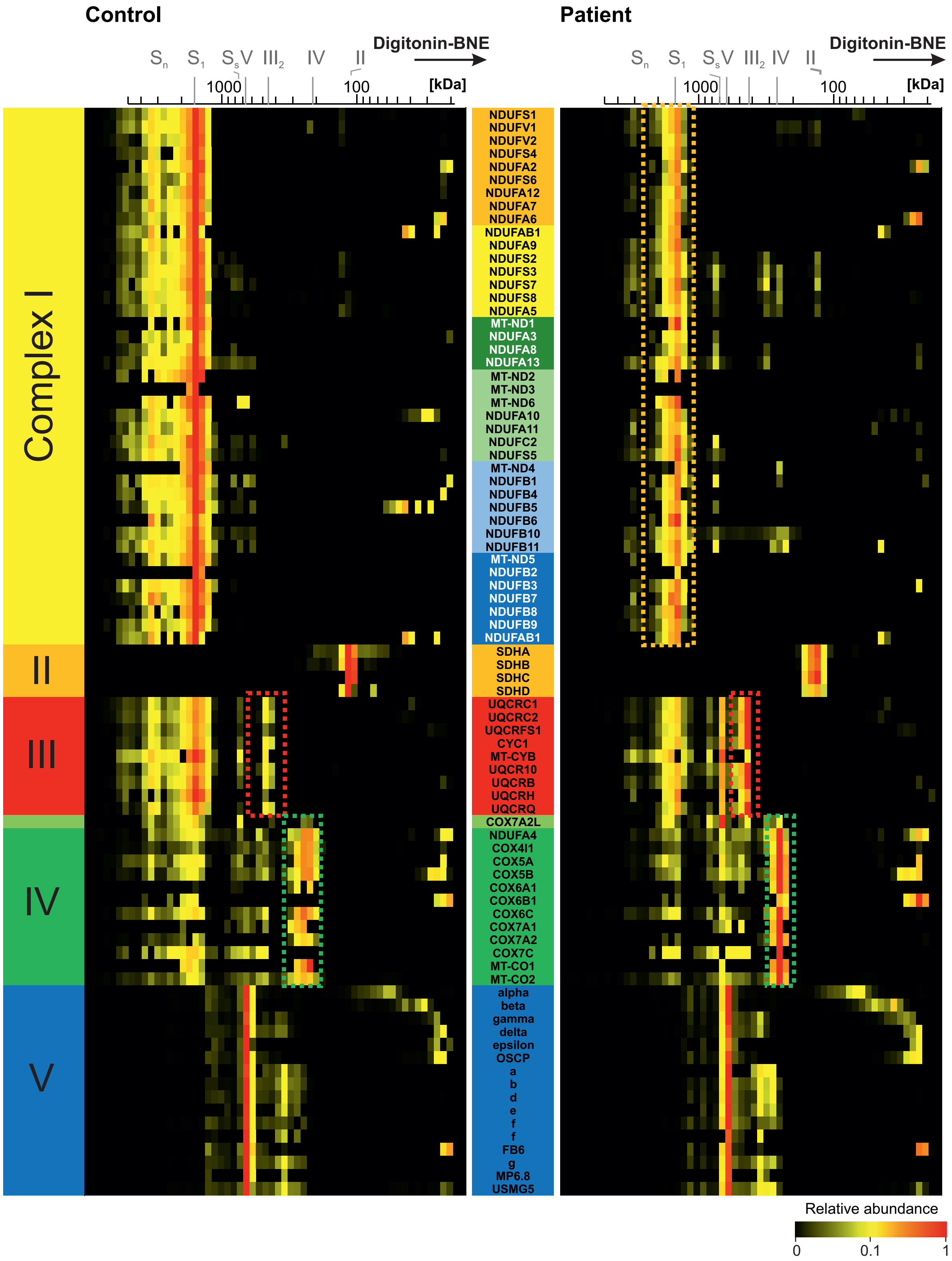

Control
Patient
Digitonin-BNE
Sn S1 Ss V III2 IV II
1000
100
[kDa]
Complex I
II
III
IV
V
NDUFS1
NDUFV1
NDUFV2
NDUFS4
NDUFA2
NDUFS6
NDUFA12
NDUFA7
NDUFA6
NDUFAB1
NDUFA9
NDUFS2
NDUFS3
NDUFS7
NDUFS8
NDUFA5
MT-ND1
NDUFA3
NDUFA8
NDUFA13
MT-ND2
MT-ND3
MT-ND6
NDUFA10
NDUFA11
NDUFC2
NDUFS5
MT-ND4
NDUFB1
NDUFB4
NDUFB5
NDUFB6
NDUFB10
NDUFB11
MT-ND5
NDUFB2
NDUFB3
NDUFB7
NDUFB8
NDUFB9
NDUFAB1
SDHA
SDHB
SDHC
SDHD
UQCRC1
UQCRC2
UQCRFS1
CYC1
MT-CYB
UQCR10
UQCRB
UQCRH
UQCRQ
COX7A2L
NDUFA4
COX4I1
COX5A
COX5B
COX6A1
COX6B1
COX6C
COX7A1
COX7A2
COX7C
MT-CO1
MT-CO2
alpha
beta
gamma
delta
epsilon
OSCP
a
b
d
e
f
f
FB6
g
MP6.8
USMG5
Relative abundance
0
0.1
1

Figure EV2. OxPhos complexes of *RTN4IP1* patient and control fibroblasts analysed by complexome profiling.

Complexome profiling data were presented as a heatmap, corresponding to subunits of individual OxPhos complexes I-V. Mitochondrial complexes were solubilised with digitonin, separated on blue-native gels (BNE), cut into fractions and analysed by quantitative mass spectrometry. Assignment of complexes: complex II (II); complex III dimer (III_2); complex IV (IV); complex V (V); small supercomplex of $CIII_2$ and CIV (S_s); supercomplex containing CI, III_2 and 1 copy of CIV (S_1) and higher order supercomlexes (S_n). The relative abundance of each protein was represented from low to high according to the colour scale illustrated on the bottom right.

 © The Author(s)

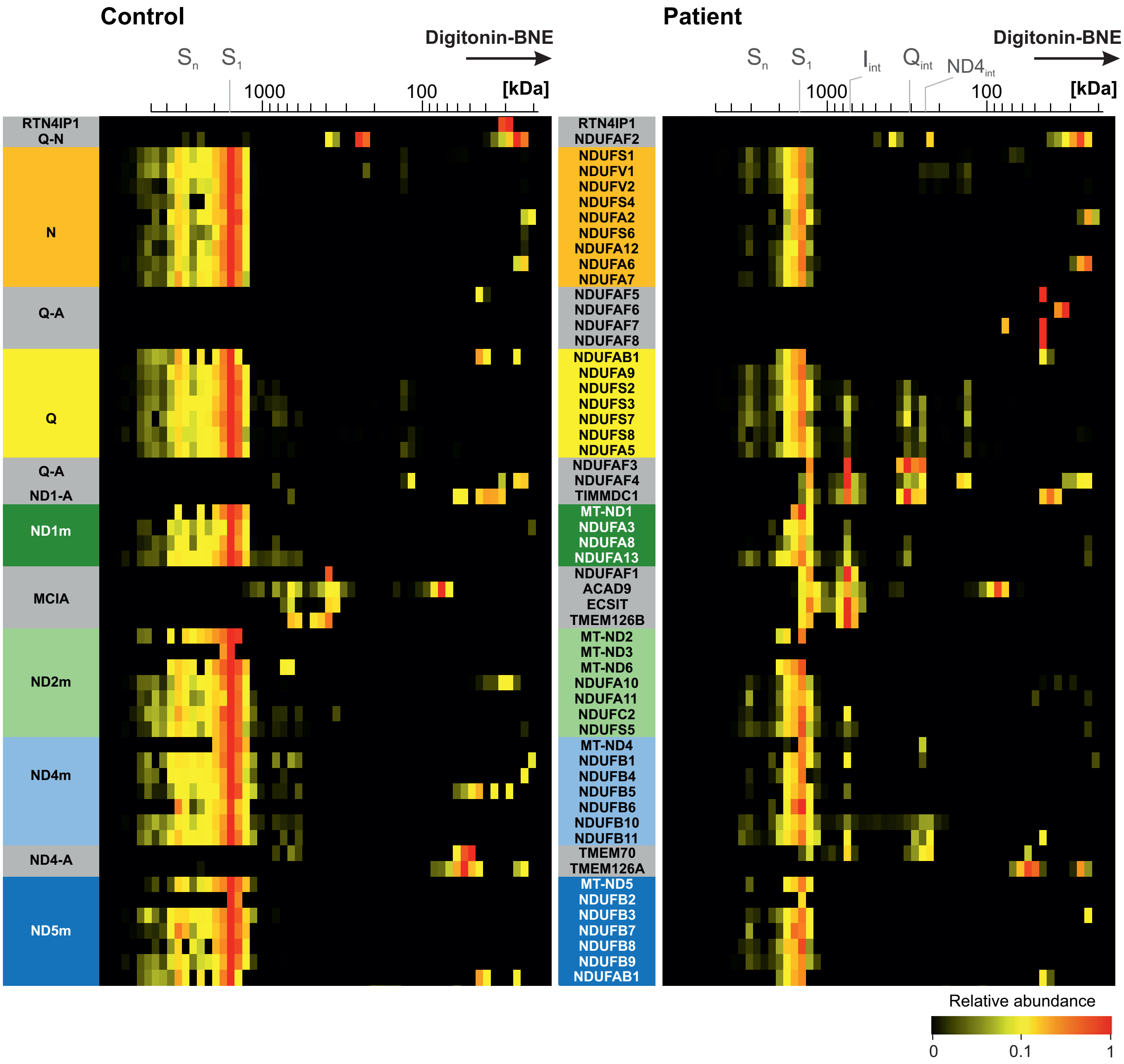

Figure EV3. CI assembly in *RTN4IP1*-derived patient fibroblasts corresponding to control cells.

Complexome profiling data of CI and identified assembly factors were sorted to their assembly modules and presented as a heatmap. Assignment of complexes: higher order supercomplexes (S_n); supercomplex S_1 of CI, $CIII_2$ and CIV (S_1); intermediate of CI including modules Q, ND1m, ND2m, ND4m and assembly factors (I_{int}); intermediate of Q-module containing assembly factors (Q_{int}) and intermediate of ND4-module containing assembly factors ($ND4_{int}$).

© The Author(s)

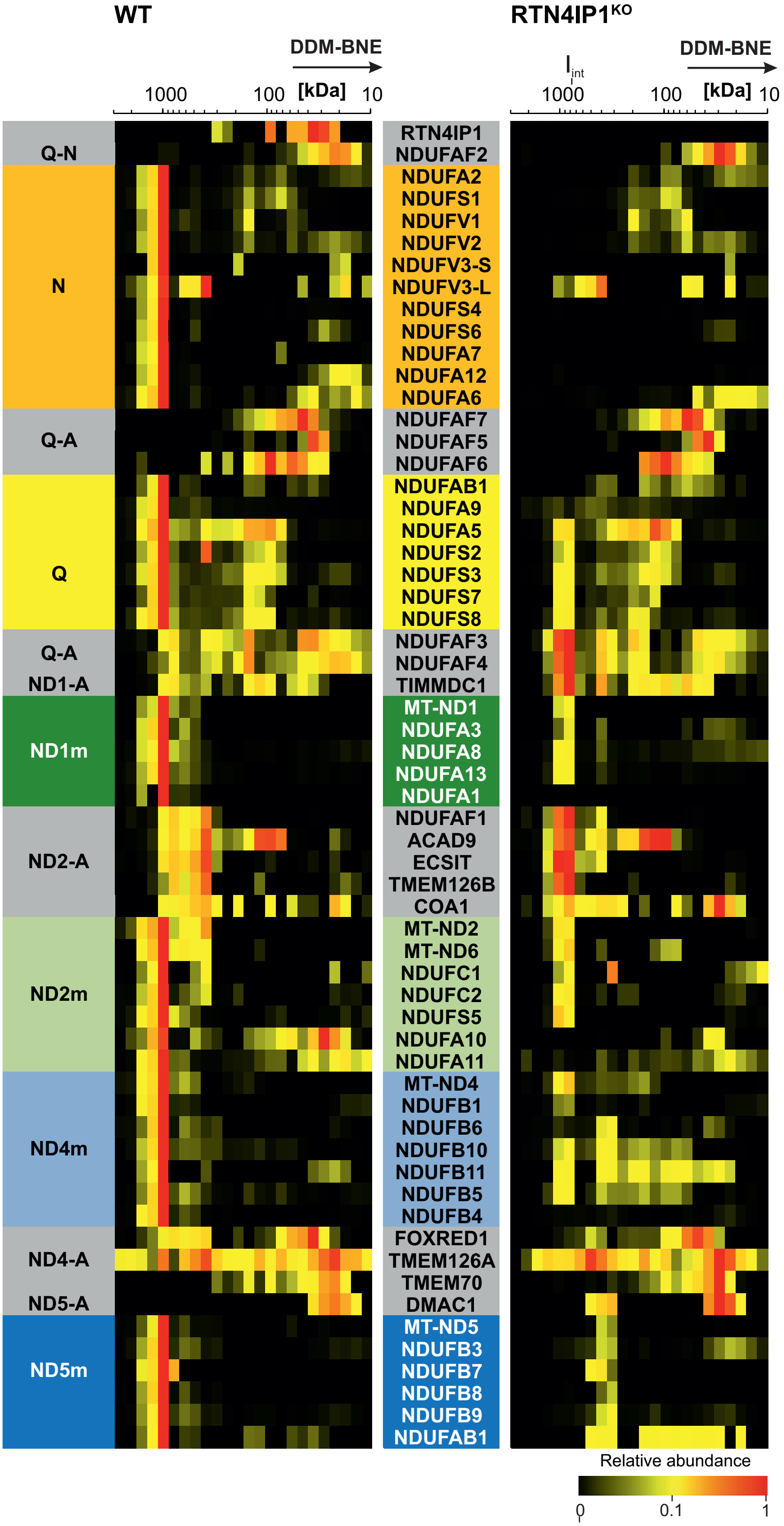
WT
RTN4IP1KO
DDM-BNE
DDM-BNE
Iint
1000
100
[kDa]
10
Q-N
N
Q-A
Q
Q-A
ND1-A
ND1m
ND2-A
ND2m
ND4m
ND4-A
ND5-A
ND5m
RTN4IP1
NDUFAF2
NDUFA2
NDUFS1
NDUFV1
NDUFV2
NDUFV3-S
NDUFV3-L
NDUFS4
NDUFS6
NDUFA7
NDUFA12
NDUFA6
NDUFAF7
NDUFAF5
NDUFAF6
NDUFAB1
NDUFA9
NDUFA5
NDUFS2
NDUFS3
NDUFS7
NDUFS8
NDUFAF3
NDUFAF4
TIMMDC1
MT-ND1
NDUFA3
NDUFA8
NDUFA13
NDUFA1
NDUFAF1
ACAD9
ECSIT
TMEM126B
COA1
MT-ND2
MT-ND6
NDUFC1
NDUFC2
NDUFS5
NDUFA10
NDUFA11
MT-ND4
NDUFB1
NDUFB6
NDUFB10
NDUFB11
NDUFB5
NDUFB4
FOXRED1
TMEM126A
TMEM70
DMAC1
MT-ND5
NDUFB3
NDUFB7
NDUFB8
NDUFB9
NDUFAB1
Relative abundance
0
0.1
1

 © The Author(s)

Figure EV4. CI assembly in U2OS WT and U2OS RTN4IP1^{KO} cells.

DDM-solubilised mitochondria complexome profiling data of CI and identified assembly factors were sorted to their assembly modules and presented as heatmap. Assignment of complexes: intermediate of CI including modules Q, ND1m, ND2m, ND4m and assembly factors (I_{int}).

© The Author(s)

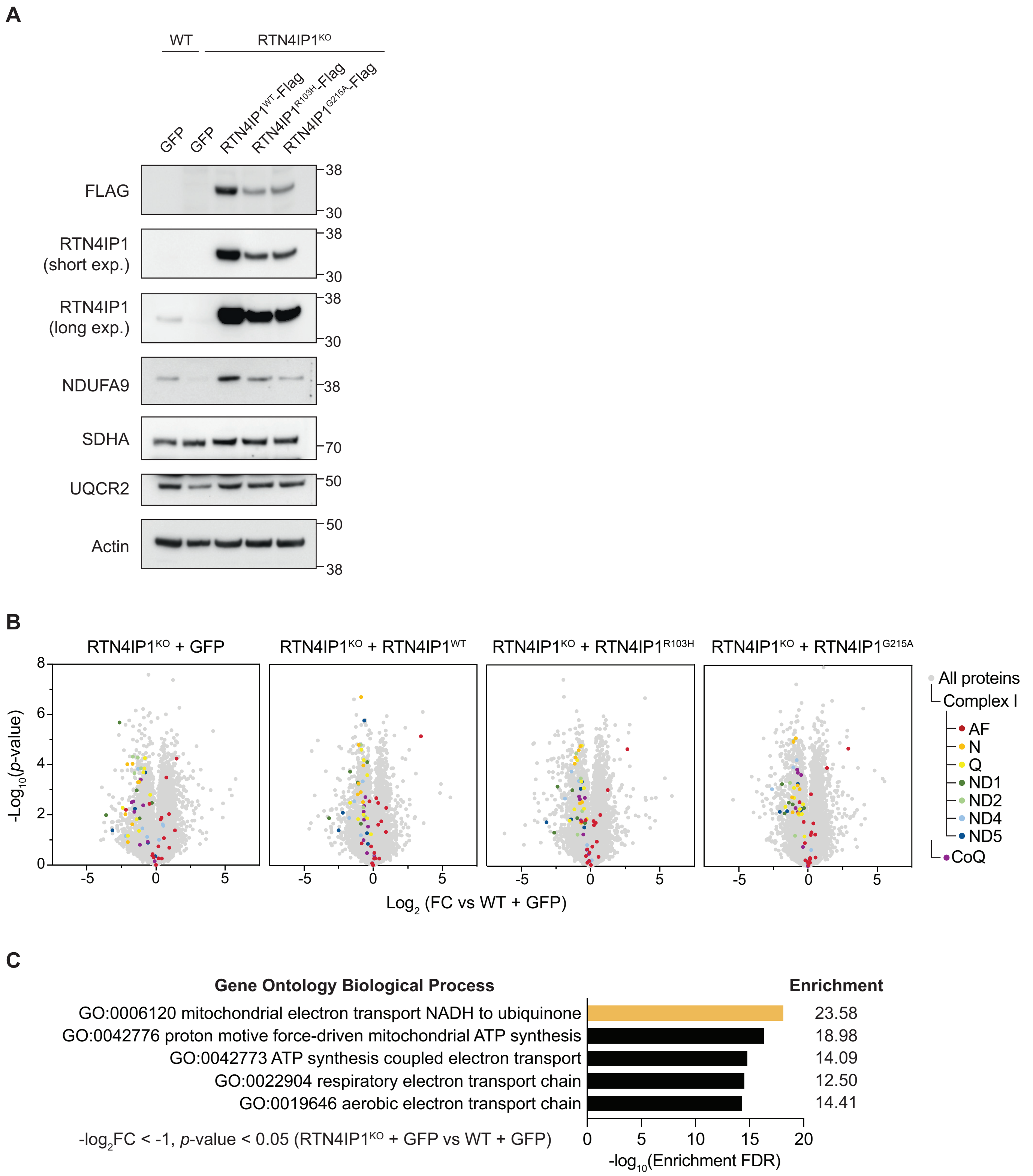
A
WT
RTN4IP1KO
GFP
GFP
RTN4IP1WT-Flag
RTN4IP1R103H-Flag
RTN4IP1G215A-Flag
FLAG
RTN4IP1 (short exp.)
RTN4IP1 (long exp.)
NDUFA9
SDHA
UQCR2
Actin
38
30
70
50
B
RTN4IP1KO + GFP
RTN4IP1KO + RTN4IP1WT
RTN4IP1KO + RTN4IP1R103H
RTN4IP1KO + RTN4IP1G215A
-Log10(p-value)
Log2 (FC vs WT + GFP)
All proteins
Complex I
AF
N
Q
ND1
ND2
ND4
ND5
CoQ
C
Gene Ontology Biological Process
Enrichment
GO:0006120 mitochondrial electron transport NADH to ubiquinone 23.58
GO:0042776 proton motive force-driven mitochondrial ATP synthesis 18.98
GO:0042773 ATP synthesis coupled electron transport 14.09
GO:0022904 respiratory electron transport chain 12.50
GO:0019646 aerobic electron transport chain 14.41
-log2FC < -1, p-value < 0.05 (RTN4IP1KO + GFP vs WT + GFP)
-log10(Enrichment FDR)

 © The Author(s)

Figure EV5. Validation of the U2OS RTN4IP1 rescue cell lines.

(**A**) Western blotting analysis validating the stable expression of RTN4IP1-FLAG constructs in the U2OS RTN4IP1^{KO} cell lines. WT and RTN4IP1^{KO} cells were infected with a GFP or FLAG-tagged RTN4IP1-encoding lentivirus, resulting in the generation of WT and RTN4IP1^{KO} cells expressing GFP, and RTN4IP1^{KO} cells expressing RTN4IP1-FLAG mutants (RTN4IP1^{WT}, RTN4IP1^{R103H} or RTN4IP1^{G215A}). Actin and SDHA were used as loading controls. (**B**) Volcano plot of proteomics experiments depicted in main Fig. 5A,B, showing protein abundances in U2OS RTN4IP1^{KO}(+*GFP*) or U2OS RTN4IP1^{KO}(+RTN4IP^{WT}, RTN4IP1^{R103H} or RTN4IP1^{G215A}) cells relative to WT(+*GFP*). CI proteins are highlighted, and colour coordinated to match individual CI modules. Data shown as mean ($n = 3$), two-sided Student's *t*-test. (**C**) Gene Ontology of Biological Process showing enrichment of mitochondrial electron transport from NADH to ubiquinone proteins based on proteomics analysis of RTN4IP1^{KO}(+*GFP*) versus WT(+*GFP*), for proteins whose $\log_2$ FC < -1 and *p value < 0.05. FDR for the relevant gene sets (top to bottom): 7.58e-19, 4.81e-17, 1.64e-15, 3.08e-15, 4.82e-15; hypergeometric test. Source data are available online for this figure.

© The Author(s)
